# Genotyping of feline leukemia virus in Mexican housecats

**DOI:** 10.1007/s00705-015-2740-4

**Published:** 2016-01-08

**Authors:** Hugo Ramírez, Marcela Autran, M. Martha García, M. Ángel Carmona, Cecilia Rodríguez, H. Alejandro Martínez

**Affiliations:** Facultad de Estudios Superiores Cuautitlán, Veterinary Medicine, Virology, Genetics and Molecular Biology Laboratory, Campus 4, Cuautitlán Izcalli Estado de México, Universidad Nacional Autónoma de México, Km 2.5 Carretera Cuautitlán–Teoloyucan, San Sebastián Xhala, CP. 54714 Cuautitlán Izcalli, Estado de México México; Immuno-Virology Laboratory, Department of Immunological Research, UMAE Pediatrics Hospital, XXI Century National Medical Center, IMSS, Av. Cuauhtémoc 330, Col. Doctores, CP. 06725 Ciudad de México, México

**Keywords:** FeLV, PCR, Phylogenetic analysis, Risk factors, Central Mexico

## Abstract

Feline leukemia virus (FeLV) is a retrovirus with variable rates of infection globally. DNA was obtained from cats’ peripheral blood mononuclear cells, and proviral DNA of *pol* and *env* genes was detected using PCR. Seventy-six percent of cats scored positive for FeLV using *env-*PCR; and 54 %, by *pol*-PCR. Phylogenetic analysis of both regions identified sequences that correspond to a group that includes endogenous retroviruses. They form an independent branch and, therefore, a new group of endogenous viruses. Cat gender, age, outdoor access, and cohabitation with other cats were found to be significant risk factors associated with the disease. This strongly suggests that these FeLV genotypes are widely distributed in the studied feline population in Mexico.

Feline leukemia virus (FeLV) belongs to the genus *Gammaretrovirus* and the family *Retroviridae*, and at least six exogenous subgroups of the virus are recognized (FeLV A, B, C, AC, D and T). These are classified according to their cellular tropism, which is mainly determined by the structural composition of the viral envelope [1]. The dominant genotype in infected cats is FeLV-A, which is the most infectious variety, but also the least virulent [2]. Genotypes FeLV-B and, particularly, FeLV-C are less common but are often present after FeLV-A infection. FeLV-B originated from the recombination of FeLV-A and endogenous viral sequences [3]. Studies using PCR have identified variable infection rates of FeLV globally. High rates of infection have been found in the United Kingdom (54 %) [4], Colombia (68 %) [5], Australia (43%) [6] and Brazil (47.5 %) [7]; intermediate rates in Spain (35.7 %) [8], Switzerland (33 %), [9] and the United States (15-20 %); and low infection rates in Canada (3-4 %) [8]. The characterization and segregation of infected cats remains the cornerstone for the prevention of new infections [10]. Gender, adulthood, access to the outdoors, and contact with other cats have all been identified as risk factors for FeLV infection, and these factors play a decisive part in the infection rate [11]. Despite the potentially fatal impact of FeLV infection in Mexican cats, very little information exists at the local and national levels [1]. The goal of this study was to identify FeLV infections and their genotypes in domestic cats in Mexico’s central region using PCR.

A heterogeneous population of 100 cats was included in the study; the cats did not present clinical signs of FeLV infection at the time of sampling (January 2012 to January 2013). The animals were found in private veterinary clinics, shelters and the veterinary hospital at the Facultad de Estudios Superiores Cuautitlán of the National Autonomous University of Mexico (FESC, UNAM). Data regarding age, gender, daily outdoor access, cohabitation with other cats, origin, and vaccination history were recorded. The study was endorsed by the FESC Internal Committee on Animal Use, Care, and Experimentation, code C13_06. Informed consent was also obtained from the owners of the cats. Blood samples were obtained by puncture of the jugular or radial veins, using tubes with anticoagulant (Vacutainer EDTA BD®, Mexico). Peripheral blood mononuclear cells (PBMCs) were purified by density gradient centrifugation. Proviral DNA was extracted from PBMCs using a commercial kit (Favorprep, FAVORGEN®, Taiwan) according to the manufacturer’s instructions. Primers for amplification of a 508-bp *env* and a 791-bp *pol* region of the FeLV genome were designed according to reference sequences using bioinformatics software [12]. The *env* primers were Fw 5′TAYTGGGCCTGTAACACYG3′ and Rv 5′CGCTGTTTTAGTCTTTCTCTTA3′, and the *pol* primers were Fw 5′CYAMCCRTTATTRGGDAGAGA3′ and Rv 5′CCAGCAAGAGGTCATCTACA3′. PCR reaction mixtures consisted of buffer 1X (Invitrogen), 1.5 mM MgCl_2_ (Invitrogen), 225 µM dNTPs (Thermo Scientific), 600 nmoles of each primer (Eurofins), 0.04 U of Platinum Taq polymerase (Invitrogen) per µl, and 1000 ng of DNA per reaction in a final volume of 30 µl. The PCR conditions were as follows: an initial denaturation step at 94 °C for 5 minutes, followed by 45 cycles at 94 °C for 1 minute, annealing at 54 °C for 60 seconds (*env* gene) or 55 °C for 45 seconds (*pol* gene), and 72 °C for 50 seconds, followed by a final elongation step at 72 °C for 10 minutes. We used DNA from both FeLV-negative and FeLV-positive cats as control material. This control DNA was previously evaluated using commercial kits (Anigen Rapid FIV Ab/FeLV Ag Test Kit). Amplification products of the anticipated size were gel-extracted using a commercial kit (FavorPrep Gel Purification Mini Kit; Favorgen Biotech Corp), and subjected to bidirectional sequencing using an API 3130x1 sequencer (genetic analyzer with 16 capillaries) at the Biotechnology and Prototype Unit of FES-Iztacala, UNAM. The obtained nucleotide sequences were edited and aligned with the BioEdit program [12]. Phylogenetic analysis of FeLV was carried out by maximum-parsimony (MP) inference. The MP tree was built using the subtree pruning and regrafting (SPR) algorithm; included codon positions were 1^st^ + 2^nd^ + 3^rd^ + noncoding. Evolutionary analysis was conducted using MEGA software version 6.06 [13]. Statistical confidence in the topology of the phylogenetic tree was secured with bootstrap values from 100 repetitions. Nodes with bootstrap values above 70 were considered significant. Trees were constructed as described by Watanabe et al. for the *env* region and Song et al. for the *pol* region [14, 15]. Genetic distances were computed using MEGA 6.06 from the nucleotide sequence alignment on the basis of the p-distance model, applying the default settings with the exception that all sites with ambiguous codes and gaps were ignored.

The characteristics of the studied cat population are shown in Table 1. Ninety-six percent of the sampled cats were not immunized; 56 % were females and 44 % were males (data not shown). Proviral DNA was detected in 76 % of the cats using *env*-PCR (Table 1), and in 54 % of the animals using *pol*-PCR. This difference in detection is probably due to the lower sensitivity of *pol*-PCR.

**Table 1:** Detection of proviral FeLV DNA in the cat population

Feature		Animal	PCR *pol* (+)^a^	PCR *env* (+)^a^
Age (years)	<1	29	16 (55 %)	22 (76 %)^**†**^
	1-3	49	24 (49 %)	39 (80 %)^**†**^
	4-9	10	6 (60 %)	6 (60 %)
	>9	12	8 (67 %)	9 (75 %)±
Gender	M	44	25 (57 %)	36 (82 %)±
	F	56	29 (52 %)	40 (71 %)
Days of outdoor access per week	1	7	3 (43 %)	4 (57 %)
	2	20	7 (35 %)	12 (60 %)±
	5	26	14 (54 %)	17 (65 %)±
	6	36	28 (78 %)±	36 (100 %)±
	Unknown	11	2 (ND)	7 (ND)
Cohabitation	0-2	16	7 (44 %)	10 (63 %)
	3-5	26	11 (42 %)	20 (77 %)±
	>5	34	26 (75 %)±	34 (100 %)±
	Unknown	24	10 (42 %)	12 (50 %)
Origin	EM	38	26 (68 %)	28 (74 %)
	MC	62	28 (45 %)	48 (77 %)
Vaccinated/FeLV	Yes	4	2 (50 %)	4 (100 %)
	No	96	52 (54%)	72 (75 %)

ND, not determined; EM, México (State); MC, Mexico City

^a^PCR (+): Number of animals FeLV positive (percent) by PCR of *pol* and *env* genes

^**±**^Age: statistical significance, *p* < 0.034*; CI: 95%; SEM 0.1233; SD: 1.345 ±

±Gender: statistical significance, *p* < 0.014*; CI: 95 %; SEM 0.879; SD 1.0567±

± Outdoor access *(pol)*: statistical significance, *p* <0.00134; CI: 95 %; SEM 1.34-2.45 ±; SD: 0.675±

± Outdoor access *(env)*: statistical significance, *p* <0.001; CI: 99 %; SEM 0.445 ±; SD: 0.045±

± Cohabitation *(pol)* statistical significance, *p* < 0.012*; CI: 96 %; SEM 0.4575; SD: 2.306 ±

± Cohabitation *(env)* statistical significance, *p* < 0.0042*; CI: 95 %; SEM 0.840; SD: 0.488±

Phylogenetic trees were constructed from the obtained nucleotide sequences deposited in the GenBank database, and are available under accession numbers KR030093 to KR030134 for *env* sequences, and KR030135 to KR030149 for *pol* sequences. In total, 42 *pol* and 10 *env* sequences were analyzed. In the tree constructed for the *env* region, the sequences generated in this study formed a new cluster of endogenous FeLV viruses with bootstrap values of 100. The sequences clustered with other branches including endogenous retroviruses (*enFeLV-GGAG,enFeLV-AGTT* and a recombinant virus *4314*; Fig. 1). In the tree representing the *pol* region, the obtained sequences also clustered with endogenous FeLV viruses (*enFeLV-GGAG,enFeLV-AGTT, Gamma 8* and *CFE-6*; Fig. 2). The different *env* sequences in this study genetically diverged from each other in the range of 0.002-0.051, and from other FeLV sequences in a range of 0.022-0.023. The *pol* sequences diverged genetically from each other in the range of 0.002-0.010, and from other FeLV sequences in the range of 0.000-0.022. A χ^2^-test was used to perform risk factor analysis. Variables with significant values (*P* < 0.005) were included in a multivariate analysis using Student’s t-test, with non-paired samples, an unbalanced design, and odds ratios (OR) (95 % confidence interval). All statistics were performed using SPSS software (version 15.0; IBM). The univariate analyses showed statistically significant results, mainly from the *env*-PCR data. FeLV prevalence was significantly higher in cats younger than three and older than nine years old (Table 1). In young cats, the lack of routine vaccination, few reproductive control practices (neutering), the lack of prevention campaigns, socialization and aggressiveness as a predominant behavior can be associated with high prevalence of infection. This was consistent with findings from other studies [16–18]. On the other hand, in cats 9 years or older, the high infection rate may be linked to the fact that most FeLV-infected cats have regressive and persistent phases due to their less-functional immune system [8, 19, 20]. The risk of FeLV infection was also associated with lifestyle, being significantly higher in cats with outdoor access (more than two days per week) compared with indoor cats and also higher in cats living with more than three other cats. Additionally, a significant difference was observed between sexes (higher rates of infection in male cats; Table 1). No associations were detected between FeLV infection and origin and vaccinated animals. During the sample period, 16 animals developed clinical signs consistent with FeLV infection: aplastic anemia, ophthalmologic disorders (Horner syndrome), ptosis, protrusion of the nictitating membrane, and lymphoma. Evidence of infection in the respiratory and digestive tracts was detected using radiology. *Env*-PCR scored positive in 94 % of cases (data not shown), thus confirming FeLV infection in these cats. It is important to mention that outdoor access and cohabitation were high risk factors for this population of sick cats.

**Fig. 1:**
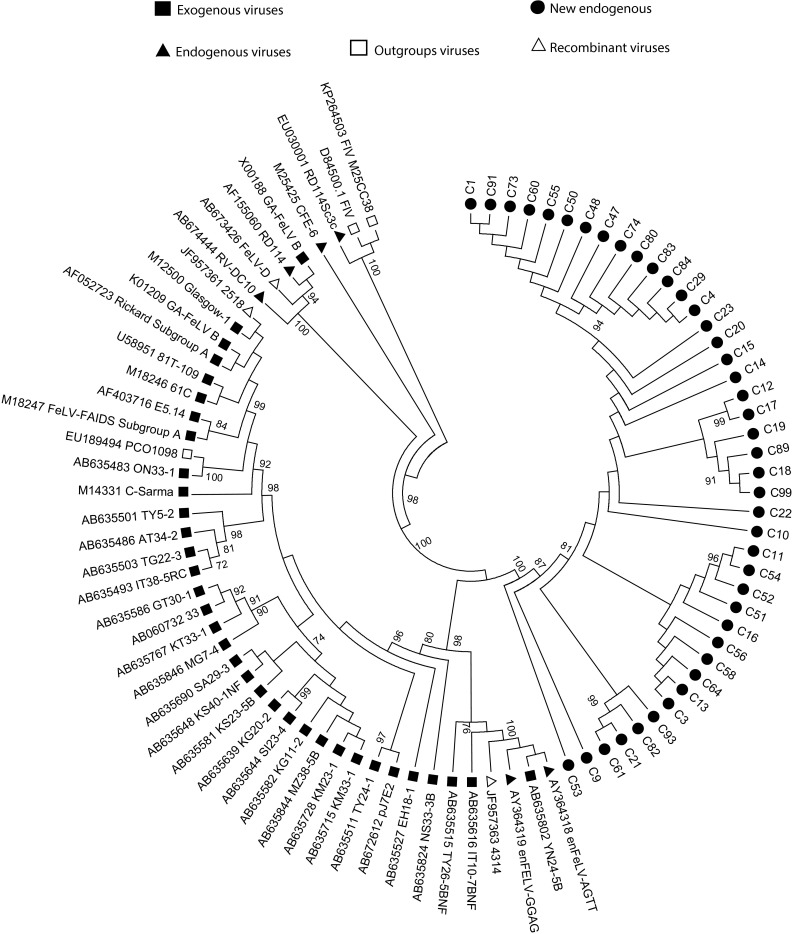
Phylogenetic tree based on the *env* region (position 7164 to 7672; envelope [SU] and transmembrane [TM] regions), including study samples and the available sequences of exogenous retrovirus (■), endogenous FeLV (▲), recombinant FeLV (∆) and outgroup viruses (□) from GenBank. The maximum-parsimony method was used for tree construction, using 100 bootstrap samples to demonstrate the robustness of groupings. The tree includes sequences described by Watanabe *et al.* [15], and accession numbers of sequences are shown. Black circles represent new endogenous FeLV

**Fig. 2:**
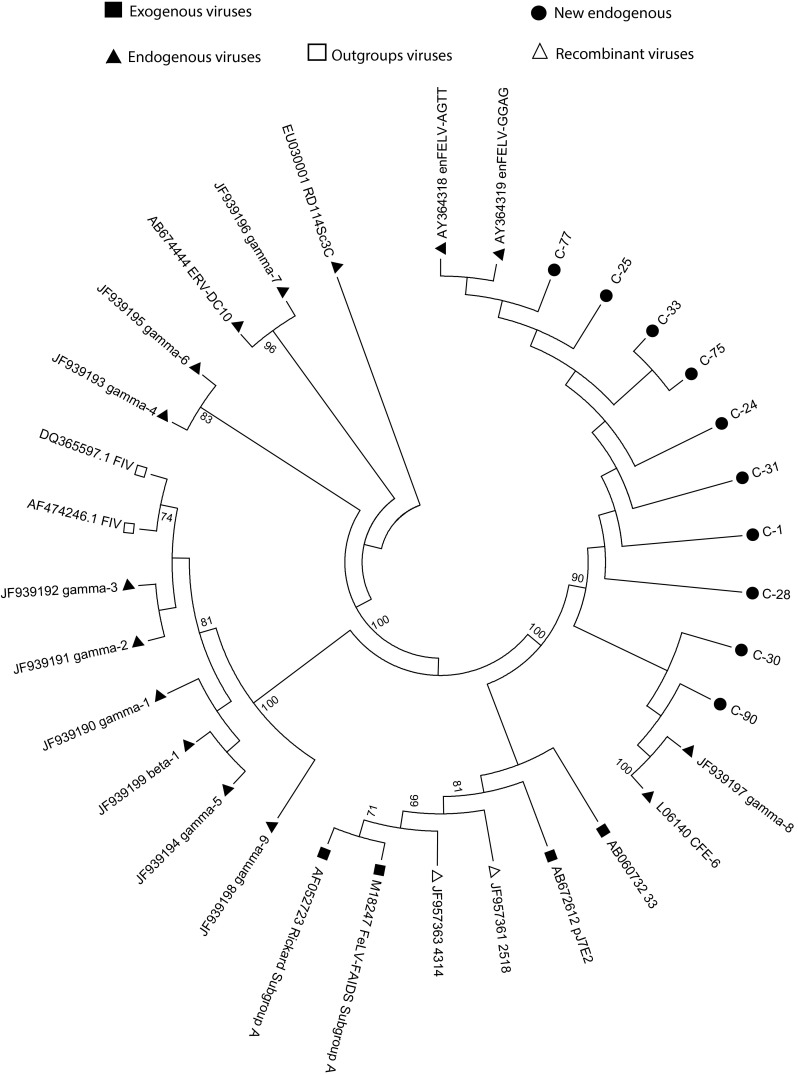
Phylogenetic tree based on the *pol* region (position 2678 to 3469; protease and reverse transcriptase regions), including study samples and the available sequences of exogenous retrovirus (■), endogenous FeLV (▲), recombinant FeLV (∆) and outgroup viruses (□) from GenBank. The maximum-parsimony method was used for tree construction, using 100 bootstrap samples to demonstrate the robustness of groupings. The tree includes sequences described by Song, *et al.* [14], and accession numbers of sequences are shown. Black circles represent new endogenous FeLV

PCR has been used in several countries to identify proviral DNA in PBMCs from infected cats [21–24]. This method is by far more sensitive than conventional immunochromatography, which can yield false negative results in suspected FeLV cases. *Env*-PCR was implemented in the present study, revealing the presence of proviral DNA in 76 % of the sampled cats. In contrast, the *pol*-PCR detection rates were lower by 22 %. This could be due to a larger number of degenerate positions in the Fw primer used to amplify the *pol* gene, thus reducing the sensitivity of the technique. We focused on amplifying fragments from the *pol* and *env* regions because the greatest genetic variability, tropism and pathogenicity are found in the *env* gene. Additionally, recombination events between both endogenous and exogenous FeLV retroviruses can involve this region [15, 25]. Likewise, the most complete characterization of endogenous FeLV was carried out for the *pol* region [14]. Endogenous viruses are important because of their interaction with exogenous FeLV and the development of clinical symptoms. The primers used for the *env*-PCR had 70 % sequence identity to exogenous viral sequences, but only sequences corresponding to endogenous viruses were identified. Phylogenetic analysis revealed that the sampled cats were only associated with endogenous FeLVs. The *env* and *pol* region phylogenetic trees showed high similarity between the sequences generated in the study and endogenous FeLVs, such as *enFeLV-GGAG*, *enFeLV-AGTT, CFE-6* and *Gamma-8*. *enFeLV-GGAG, enFeLV-AGTT* and endogenous FeLV CFE-6 have been associated with the development of clinical illness in cats [19]. These viruses are generated through the recombination of the FeLV-A genotype and endogenous *envFeLV* [26]. However, while sequences related to endogenous FeLV were identified in the sampled cats, sequences related to FeLV-A were not. It was initially considered impossible to link the development of clinical symptoms with infection by endogenous retroviruses, since their genomes are generally interrupted by stop codons, deletions, or mutations in the reading frame [25]. However, it has since been demonstrated that some endogenous retroviruses are transcriptionally active and that it is possible to find development of viral particles by infection from endogenous retroviruses. These facts may explain the link between disease development and the presence of endogenous FeLV observed in 16 of the sampled cats.

In our study, one of the observed risk factors was frequent (weekly) outdoor access, which was associated with an increase in the detection of infected individuals (in both males and females). In addition to outdoor access and cohabitation, population density and overpopulation promote stress and bad hygiene due to direct contact among cats [18, 26]. Similar results have been found in other studies that evaluated the risk factors associated with gender, age, outdoor access, and cohabiting with another cats [1, 27–29]. Other studies have demonstrated that non-neutered males have increased susceptibility and frequency of FeLV infection [18, 20]. This type of infection has also been described as being favored by factors such as outdoor access and cohabitation with more than three other cats. It has been shown that males run a higher risk of infection than females (82 % vs. 71 %) [26].

Our results demonstrated high prevalence of FeLV in cats from central Mexico, and the significant influence of risk factors such as the lack of prophylactic schemes, age, behavior and cohabitation, as elements determining FeLV infection. Additional studies are needed to reveal the pathogenic role of endogenous FeLV in central Mexican felines to evaluate their role in protection, tropism and possible interference with exogenous FeLV. Although a wide phylogenetic diversity was observed among the sequences available in the GenBank database and those generated in this study, no association was found with any sequence derived from exogenous retroviruses, even when taking into account that they are considered widely distributed and that they have been described on multiple continents. This is especially true for the FeLV-A genotype, which is mainly responsible for transmission among domestic cats [8, 30]. Although there is research on FeLV prevalence in Mexico, no other studies of genotyping have been performed. This could identify new endogenous FeLV in the central Mexican population of domestic cats that show a close relationship to other endogenous FeLV described in the GenBank database.
